# PD-L1 expression, tumor mutational burden, and immune cell infiltration in non-small cell lung cancer patients with epithelial growth factor receptor mutations

**DOI:** 10.3389/fonc.2022.922899

**Published:** 2022-08-05

**Authors:** Tiantian Ma, Jin Jiao, Ran Huo, Xiaofang Li, Guotao Fang, Qi Zhao, Weiwei Liu, Xiao Han, Chenglin Xi, Yanan Wang, Yanhong Shang

**Affiliations:** ^1^ Department of Medical Oncology, Affiliated Hospital of Hebei University, Hebei Key Laboratory of Cancer Radiotherapy and Chemotherapy, Baoding, China; ^2^ Department of Pathology, Affiliated Hospital of Hebei University, Baoding, China

**Keywords:** Non-small cell lung cancer, Epidermal growth factor receptor, Tumor immune microenvironment, Tumor mutational burden, Programmed death-ligand 1

## Abstract

**Background:**

Immunotherapy using programmed cell death protein 1/programmed death-ligand 1 (PD-1/PD-L1) inhibitors seems less effective in non-small cell lung cancer (NSCLC) patients with epithelial growth factor receptor (EGFR) mutations. Varied responses to PD-1/PD-L1 inhibitors have recently been observed in NSCLC patients harboring different types of EGFR mutations. Some EGFR-mutated NSCLC patients may benefit from PD-1/PD-L1 inhibitors. At present, PD-L1 expression, tumor mutational burden (TMB), and tumor immune microenvironment (TIME) are biomarkers for predicting the efficacy of PD-1/PD-L1 inhibitors in NSCLC patients. We retrospectively evaluated PD-L1 expression, TMB, and immune cell infiltration in NSCLC patients with EGFR mutation subtypes.

**Methods:**

PD-L1 expression, TMB, and the abundance of immune cell infiltration in NSCLC patients were evaluated in public databases and clinical samples. TMB was detected using the NGS technique, PD-L1 was detected using immunohistochemistry, and the abundance of immune cell infiltration in NSCLC samples was detected using multiple immunohistochemistry.

**Results:**

PD-L1 expression and TMB were lower in EGFR-mutated NSCLCs than in wild-type patients. Differences in the abundance of immune cell infiltration were also observed between EGFR-mutated and wild-type NSCLC. The expression of PD-L1, TMB, and abundance of immune cell infiltration were different in patients harboring different subtypes of EGFR mutations. Patients with uncommon EGFR mutations, especially the G719X mutation, showed higher TMB and expressions of PD-L1 than classical EGFR mutations. M1 macrophages were higher in uncommon EGFR mutations than classical EGFR mutations.

**Conclusions:**

The expression of PD-L1 and TMB in uncommon EGFR-mutated NSCLCs, especially the G719X mutation, were higher than those for classical EGFR-mutated NSCLCs and similar to EGFR wild-type. The abundance of immune cell infiltration in uncommon EGFR-mutated NSCLCs was similar to that in EGFR wild-type. Our findings suggest that uncommon EGFR-mutated NSCLCs may benefit from PD-1/PD-L1 inhibitors.

## Introduction

Lung cancer is the leading cause of cancer-related death around the world (1). Non-small cell lung cancer (NSCLC) accounts for 85% of all lung cancer types, of which lung adenocarcinoma, the most common subtype of NSCLC, accounts for 30-35% of total lung cancer cases (2). There were approximately 50% of Asians and 10-15% of Caucasians with lung adenocarcinoma harboring mutations in epithelial growth factor receptor (EGFR) gene (3). Over decades, EGFR tyrosine kinase inhibitors (TKIs) were developed from first to third generations and significantly improved progression-free survival (PFS) and overall survival (OS) in patients with EGFR-sensitive mutations (4–10). However, drug resistance will occur in most patients. Recently, immune checkpoint inhibitors using programmed cell death protein 1/programmed death-ligand 1 (PD-1/PD-L1) inhibitors have been used for NSCLC. However, many studies have indicated that PD-1/PD-L1 inhibitors used in EGFR-TKI-resistant NSCLC patients were not as effective as chemotherapy, with an increased rate of immune-related side effects (11–14). In the past, clinical studies for first-line immunotherapies in EGFR-mutated NSCLC patients have been terminated due to poor efficacy and obvious side effects. Therefore, EGFR-mutated NSCLC patients were restricted for clinical studies and practice using immunotherapy.

Following resistance to EGFR-TKI, subgroup analyses of an impower150 study indicated that EGFR-mutated NSCLC patients might benefit from the combination of Atezolizumab, Bevacizumab, Carboplatin, and Paclitaxel (15). Subsequent studies have shown that some subgroups of EGFR-mutated NSCLC patients may benefit from immune checkpoint inhibitors (15–19). Yamada et al. (19) found that the use of immune checkpoint inhibitors significantly improved PFS in patients with uncommon EGFR mutations than classic mutations (L858R, ex19del). Until now, there is a lack of large-scale clinical studies on the efficacy of immune checkpoint inhibitors in NSCLC patients with different subtypes of EGFR mutations. PD-L1, tumor mutational burden (TMB), and tumor immune microenvironment (TIME) are considered to be biomarkers for predicting the efficacy of immune checkpoint inhibitors in NSCLC. However, the use of these biomarkers in NSCLC patients with different subtypes of EGFR mutations remains undefined. In this study, we performed biological information analyses, next-generation sequencing (NGS) detection, immunohistochemistry (IHC), and multiple immunohistochemistry to evaluate PD-L1, TMB, and immune cell infiltration in subtypes of EGFR-mutated NSCLC. The study provides a theoretical reference for selecting EGFR-mutated NSCLC patients that may benefit from immune checkpoint inhibitors.

## Materials and methods

### Sample and data collection

Network dataset: A dataset containing RNA-seq gene expression profiles, gene mutation data, and the clinical data of NSCLC patients was downloaded from The Cancer Genome Atlas (TCGA) dataset (https://portal.gdc.cancer.gov/). Gene mutation information for NSCLC patients within the TCGA dataset was downloaded from the cBio Portal Dataset (http://www.cbioportal.org/). A protein dataset of NSCLC patients was downloaded from the TCPA dataset (https://www.tcpaportal.org/tcpa/index.html). Data from 474 NSCLC patients were analyzed.

Clinical validation dataset: Between January 2012 and October 2020, a total of 1,111 NSCLC patients, including 442 patients with an EGFR wild-type and 669 patients with EGFR alterations, were recruited at the Affiliated Hospital of Hebei University. Thirty-three patients with qualified specimens in the EGFR mutation population were selected, and their specimens were used to detect the abundance of immune cell infiltration within the tumor microenvironment. Clinical data were obtained from an electronic medical records database, and all patients provided written informed consent for the use of their tumor specimens. The study protocol was approved by the ethics committee of the Affiliated Hospital at Hebei University.

### Calculation of TMB and immune cell infiltration

TMB is defined as the sum of somatic/acquired mutations in each gene coding region of tumors and is also considered to be the total number of base mutations per megabase. Gene mutation information for NSCLC samples was downloaded from the TCGA dataset. After excluding silent mutations, the TMB value of each sample was calculated using the R language. Four hundred and fifty (450) oncogenes in each clinical sample were additionally detected using NGS technology based on NovaSeq6000 (Illumina, CA, USA), and the TMB of each sample was calculated.

The mRNA of each sample within the TCGA database was downloaded and collated, and the content of immune cells in each tumor sample was calculated using the CIBERSORT algorithm.

### Immunohistochemistry and multiple immunohistochemistry

Tumor tissues from the core biopsies of resected samples were used to perform IHC testing. Immunohistochemical staining for PD-L1 was performed using Dako 22C3 (Agilent, California, USA). The test was performed using EnVision FLEX visualization system on the DAKO Autostainer Link 48. PD-L1 IHC 22C3 pharmDx kit and EnVision FLEX washing buffer (20X) used in the experiment were purchased from Mercado Co., Ltd (China). A minimum of 100 viable tumor cells (TCs) must be present for evaluation.

Samples that provided ≥ 5 sections and samples with a proportion of malignant tumor cells ≥ 30% were selected for multiple immunohistochemical testing, which was used to detect the infiltration of immune cells (CD8 + T cells, CD3 + T cells, M1 and M2 macrophages, NK cells, CD3 + PD1 + T cells, CD3 + CD8 + T cells, and CD8 + PD1 + T cells) in tumor samples. PANO 7-plex IHC kit (Panovue, Beijing, China) was used for multiplex immunohistochemical staining. Different primary antibodies were successively applied, and then horseradish peroxidase-coupled secondary antibody incubation and tyrosamine signal amplification (TSA) were performed. After each TSA operation, the slide was subjected to microwave heat treatment. After all human antigens were labeled, the nuclei were stained with 4 ‘ -6 ‘ -diamino-2-phenylindole (DAPI). The stained slides were scanned using the Mantra System (PerkinElmer, Waltham, Massachusetts, US) to obtain multispectral images, and a single stack image is established by combining the scanning. The autofluorescence spectra of tissues and fluoresceins were extracted from the images of unstained and single-stained sections, respectively. Through inForm image analysis software (PerkinElmer, Waltham, Massachusetts, US), the extracted images were further used to build the spectral library required for multispectral separation. Using this spectral library, we obtained slice reconstructions with autofluorescence removed. All stained tissues were independently scored by two pathologists who were blinded to the clinical parameters.

### Evaluating index

Two board-certified pathologists independently evaluated all stained slides for PD-L1 staining. Per the reported standard recommendation (20, 21), the PD-L1 tumor proportion score (TPS), the percentage of TCs showing partial or complete membrane staining, was calculated. We divided samples into three groups according to TPS: < 1%, 1-49%, and ≥ 50%. We identified positive PD-L1 expression using a cut-off of ≥ 1%. High tumor mutation load (TMB-H) was defined as TMB ≥ 10 mutations/Mb.

PD-1 expression on TCs and CD8 + T cell infiltration was defined as PD-1+/CD8+. PD-1 expression on TCs and CD3 + T cell infiltration was defined as PD-1+/CD3+. CD3 + T and CD8 + T cell infiltration was defined as CD3+/CD8+. CD68+CD163- cells were defined as a M1 macrophage. CD68+CD163+ cells were defined as a M2 macrophage. CD56+ cells were defined as NK cells.

### Statistical analyses

Statistical analyses were performed using SPSS, Version 24.0, for clinical samples, and R, Version 4.0.2, for downloaded data. A chi-square test or the Fisher exact probability method was used for analyzing the clinical characteristics of EGFR-mutated NSCLC patients. A Wilcoxon rank sum test was used for comparing differences in PD-L1 expression, TMB, and the abundance of immune cell infiltration between EGFR wild-type patients and EGFR-mutated patients. All results were tested using a bilateral P test, and P ≤ 0.05 was considered statistically significant.

## Results

### Patient characteristics within the TCGA database

Clinical information for 474 NSCLC patients extracted from the TCGA dataset was analyzed (**Supplementary Tables 1**, **2**). In our study, there were 423 patients (423/474, 89.2%) with EGFR wild-type and 51 patients (51/474, 10.8%) harboring EGFR alterations. In EGFR-mutant patients, 17 patients possessed classical EGFR mutations (17/51, 3.6%), 17 patients possessed uncommon EGFR mutations (17/51, 3.6%), and 17 patients possessed EGFR amplification (17/51, 3.6%). No statistically significant differences were observed in gender, age, or TNM (The Classification of Malignant Tumors) stage between patients with EGFR alteration and wild-type (P > 0.05).

### Patient characteristics within clinical center data

**Figure 1:**
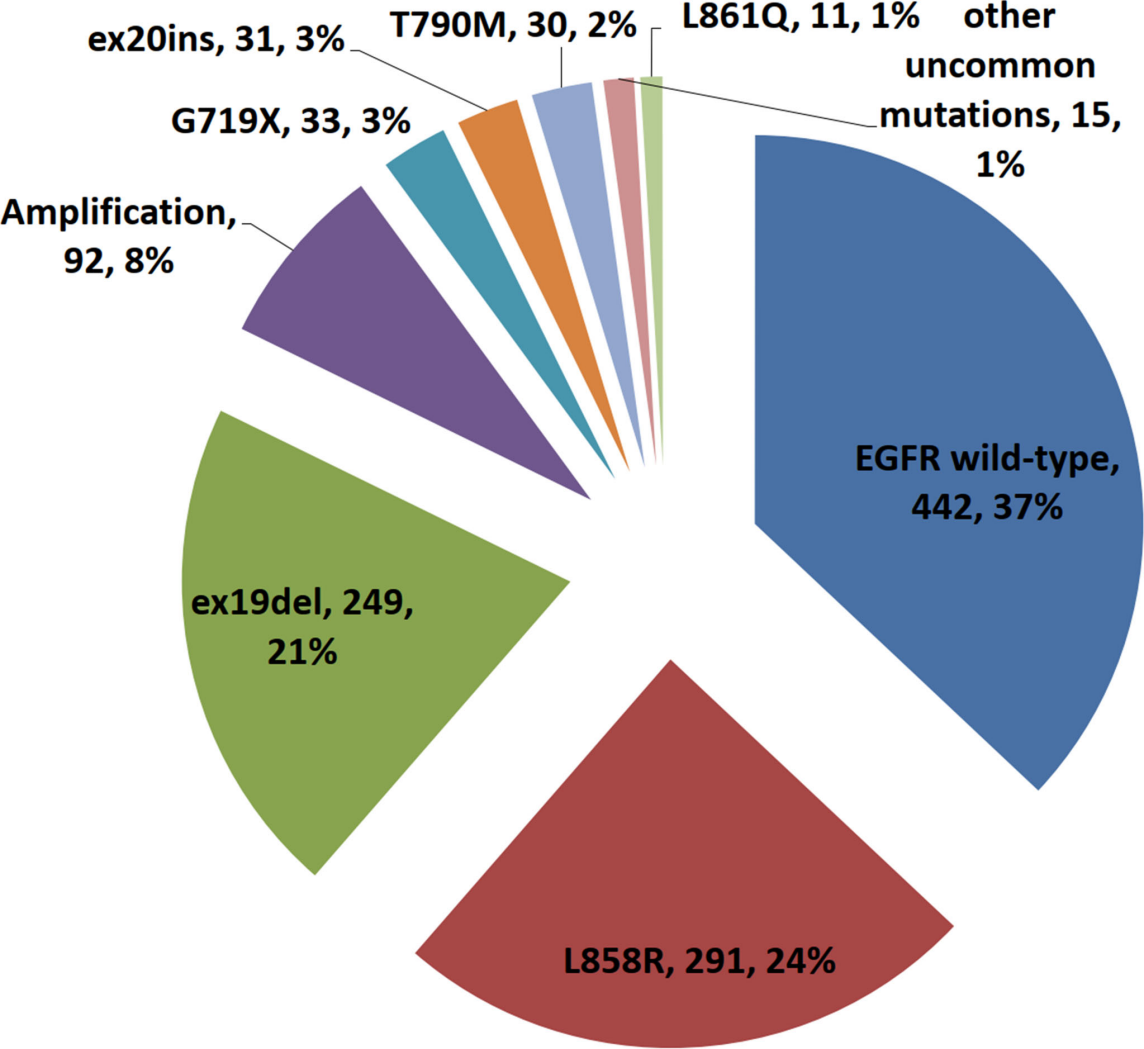
The proportion of patients with different subtypes of EGFR mutations in the 1,111 NSCLCs.

A total of 1,111 NSCLC patients were enrolled in our clinical center, including 442 patients with EGFR wild-type and 669 patients with EGFR alterations. The presence of L858R mutation and ex19del mutation were the two most frequent mutations (**Figure 1**). The summary of demographic and clinic information of the 1,111 patients was listed in **Table 1**. There were 547 males (49.2%, 547/1,111) and 564 females (50.8%, 564/1,111). EGFR mutation showed higher frequency in female, non-smoking, and stage I NSCLC patients (P < 0.001). Compared with EGFR wild-type group, there were less patients in EGFR-mutated group showed TMB-H (TMB ≥ 10 mutations/MB, 11.1% vs. 37.6%, P < 0.001) and high expression of PD-L1 (TPS: ≥ 50%, 4.8% vs. 11.8%, P < 0.001).

**Table 1:** Baseline characteristics for the 1,111 NSCLC patients.

Characteristics	Total (N = 1,111)	EGFR-WT (N = 442)	EGFR-alteration (N = 669)	P-value
Age (median, IQR)	---	61, 53-68	59, 53-67	0.251
Gender				< 0.001
Male	547 (49.2%)	296 (67.0%)	251 (37.5%)	
Female	564 (50.8%)	146 (33.0%)	418 (62.5%)	
Stage				0.001
I	414 (37.3%)	135 (30.5%)	279 (41.7%)	
II	86 (7.7%)	36 (8.1%)	50 (7.5%)	
III	145 (13.1%)	74 (16.7%)	71 (10.6%)	
IV	360 (32.4%)	149 (33.7%)	211 (31.5%)	
Unknown	106 (9.5%)	48 (10.9%)	58 (8.7%)	
Smoking				< 0.001
No	655 (59.0%)	208 (47.0%)	447 (66.8%)	
Yes	287 (25.9%)	159 (36.0%)	128 (19.1%)	
Unknown	169 (15.2%)	75 (17.0%)	94 (14.1%)	
TMB (mutations/Mb) (median, IQR)	---	6.2, 2.5-14.4	3.7, 1.8-6.1	< 0.001
TMB ≥ 10 mut/Mb	240 (21.6%)	166 (37.6%)	74 (11.1%)	
TMB < 10 mut/Mb	871 (78.4%)	276 (62.4%)	595 (88.9%)	
PD-L1				< 0.001
< 1%	841 (75.7%)	295 (66.7%)	546 (81.6%)	
1-49%	186 (16.7%)	95 (21.5%)	91 (13.6%)	
≥ 50%	84 (7.6%)	52 (11.8%)	32 (4.8%)	

A total of 33 EGFR-mutated NSCLC patients were identified from 669 patients with EGFR alterations (**Supplementary Table 3**). There were 30.3% (10/33) patients possessed uncommon EGFR mutations; and 69.7% (23/33) patients possessed classical EGFR mutations. No statistically significant differences were observed in gender, age, stage, operation, smoking status, and pathological type between patients with uncommon and classical EGFR mutations (P > 0.05).

### The expression of PD-L1 in EGFR mutated NSCLC patients

**Figure 2:**
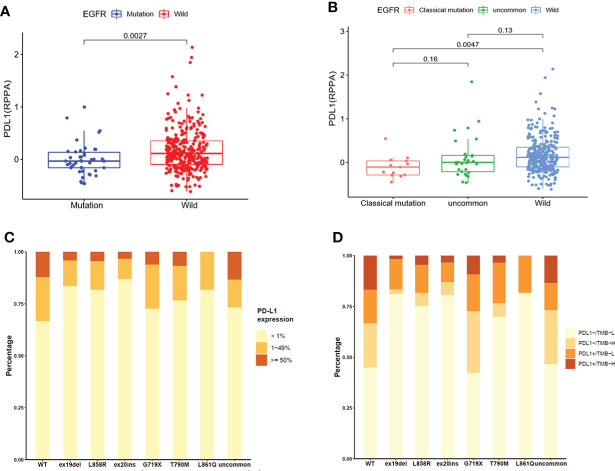
The expression of PD-L1 in TCGA database **(A, B)** and clinical center **(C, D)**. **(A)** Comparison of PD-L1 expression in NSCLC patients from TCGA database between EGFR wild-type and EGFR-mutated groups. **(B)** Comparison of PD-L1 expression in NSCLC patients from TCGA database between EGFR classical mutations, uncommon mutations, and EGFR wild-type groups. **(C)** The expression of PD-L1 in patients with different EGFR mutations in our clinical center. **(D)** The distribution of PD-L1-/TMB-L, PD-L1-/TMB-H, PD-L1+/TMB-L, and PD-L1+/TMB-H in NSCLC patients with different EGFR mutations in our clinical center.

A total of 355 NSCLC samples in TCGA database were tested for PD-L1 expression. We compared the expression of PD-L1 in NSCLC patients between EGFR wild-type and EGFR-mutated groups (**Figure 2A**) and observed higher expression in EGFR wild-type patients (P = 0.0027). We further compared the expression of PD-L1 in patients with different subtypes of EGFR mutations and EGFR wild-type (**Figure 2B**). The expression of PD-L1 in patients with uncommon EGFR mutations was comparable to EGFR wild-type (P = 0.13), while the expression of PD-L1 in patients with classical EGFR mutations was lower as compared to EGFR wild-type (P = 0.0047). Although the median value of PD-L1 in patients with uncommon EGFR mutations displayed a higher trend as compared to classical EGFR mutations, the difference was not statistically significant (P = 0.16).

The expression of PD-L1 in subgroups of patients with EGFR mutations in our clinical center were provided in **Figure 2C**. The positive expression of PD-L1 in patients with uncommon EGFR mutations (G719X, L861Q, and other rare mutations) were higher than that in patients with classical EGFR mutations (19del, L858R, T790M). Patients were divided into four groups as PD-L1-/TMB-L, PD-L1-/TMB-H, PD-L1+/TMB-L, and PD-L1+/TMB-H. The distribution of TMB and PD-L1 in EGFR mutated NSCLC patients were different within different EGFR mutation subtypes (**Figure 2D**). The proportion of patients with PD-L1+/TMB-H in G719X and other uncommon EGFR mutations was higher than that in other subtypes of EGFR mutations (P < 0.001).

### TMB in EGFR mutated NSCLC patients

**Figure 3:**
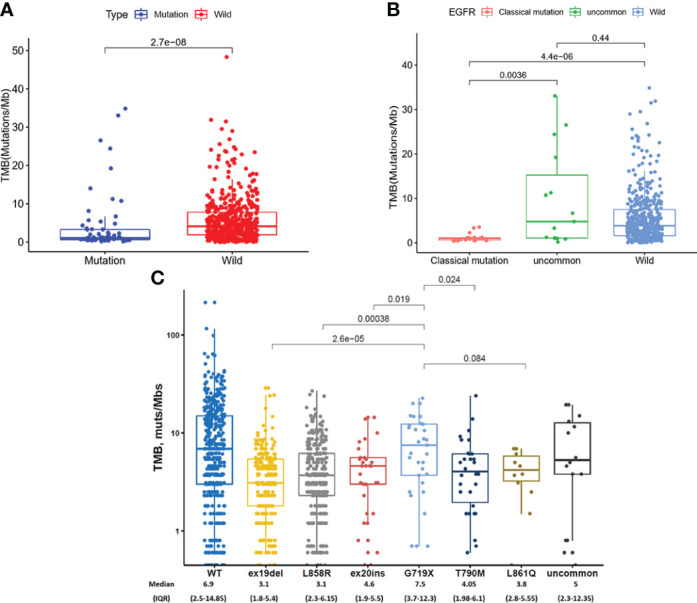
TMB in TCGA database **(A, B)** and clinical center **(C)**. **(A)** Comparison of TMB in NSCLC patients from TCGA database between EGFR wild-type and EGFR-mutated groups. **(B)** Comparison of TMB in NSCLC patients from TCGA database between EGFR classical mutations, uncommon mutations, and EGFR wild-type groups. **(C)** The expression of TMB in patients with different EGFR mutations in our clinical center.

We analyzed TMB in EGFR mutated NSCLC patients in TCGA database and found that TMB in EGFR wild-type patients was higher than that in EGFR mutated patients (P = 2.7 × 10^-8^, **Figure 3A**). As shown in **Figure 3B**, TMB was lower in patients with classical EGFR mutations than uncommon EGFR mutations (P = 0.0036) and EGFR wild-type (P = 4.4 x 10^-6^). TMB between patients with uncommon EGFR mutations and EGFR wild-type were comparable (P = 0.44).

In our clinical center, the highest TMB level was observed in patients with a G719X mutation, which were higher than ex19del (7.5 vs. 3.1 mutations/Mb, P < 0.001), L858R (7.5 vs. 3.4 mutations/Mb, P < 0.001), ex20ins (7.5 vs. 4.6 mutations/Mb, P = 0.019), and T790M (7.5 vs. 4.05 mutations/Mb, P = 0.024) (**Figure 3C**).

**Figure 4:**
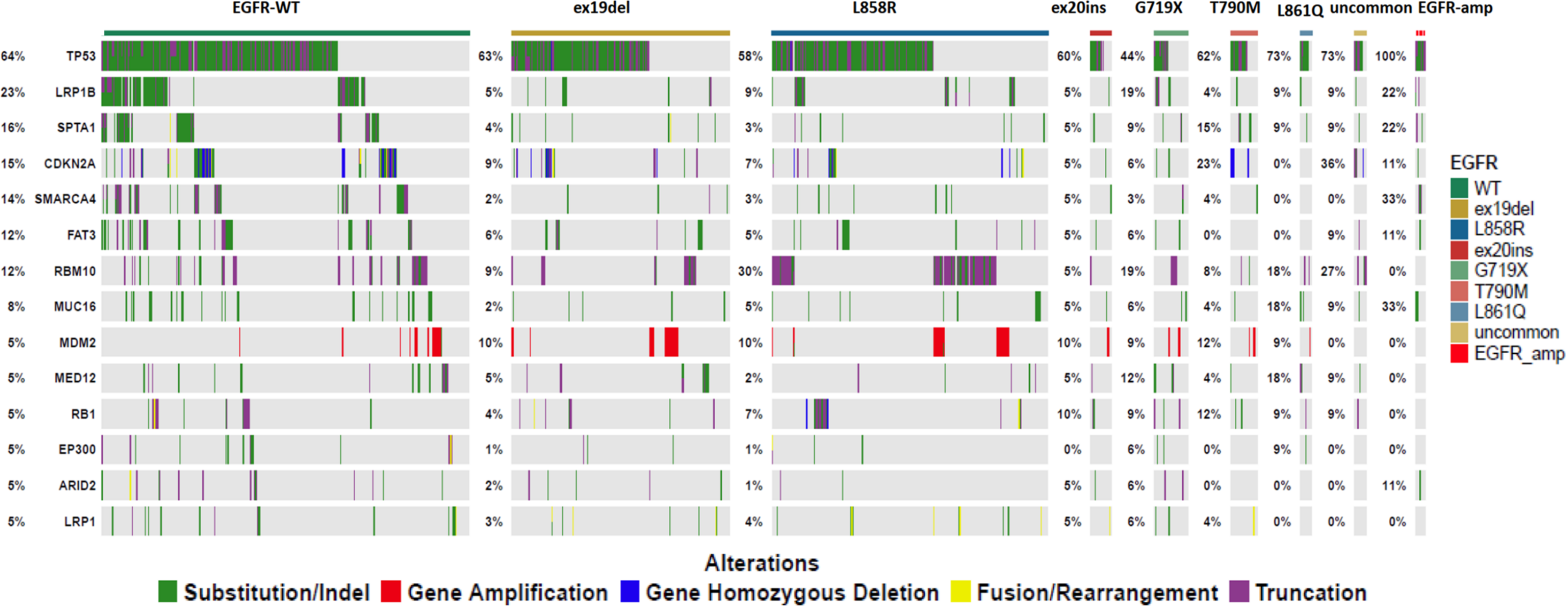
The co-mutation genetic alteration heatmap for different subtypes of EGFR mutations.

To investigate the effect of other gene mutations on EGFR mutation samples, we detected co-mutation genes with EGFR mutation subtypes (**Figure 4**). TP53 was a high-frequency, co-mutated gene for all subtypes of EGFR mutations. LRP1B were more frequently co-mutated with G719X mutation than other EGFR mutations.

### Immune cell infiltration in EGFR mutated NSCLC patients

**Figure 5:**
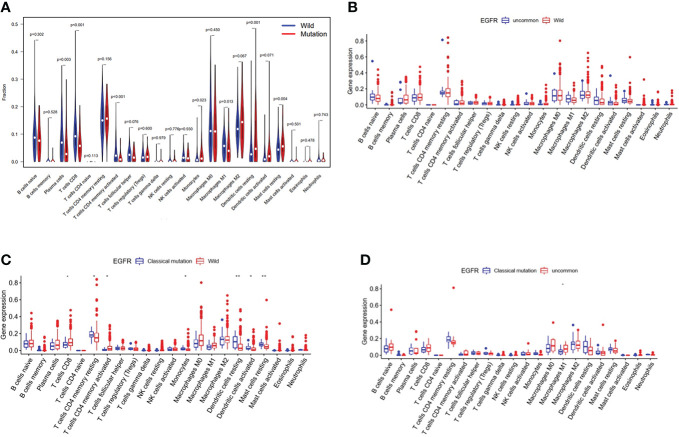
Immune cell infiltration in TCGA database. Comparison of immune cells in NSCLC patients from TCGA database between EGFR wild-type and EGFR-mutated groups **(A)**, EGFR uncommon mutations and EGFR wild-type groups **(B)**, EGFR classical mutations and EGFR wild-type groups **(C)**, and EGFR classical mutations and uncommon mutations **(D)**.

Immune cell infiltration in EGFR mutated NSCLC patients in TCGA database were shown in **Figure 5**. The violin map of immune cell infiltration indicated that the infiltration of CD8 + T cells (P < 0.001), activated CD4 + T cells (P < 0.001), and M1 macrophages (P = 0.013) were lower in EGFR mutated patients than wild-type (**Figure 5A**). The comparison of immune cell infiltration observed non-significant difference between patients with uncommon EGFR mutations and EGFR wild-type (**Figure 5B**). The infiltration of CD8 + T cells and activated CD4 + T cells was lower in patients with classical EGFR mutations than wild-type (P < 0.05) (**Figure 5C**). The infiltration of M1 macrophages was higher in patients with uncommon EGFR mutations than classical EGFR mutations (P < 0.05) (**Figure 5D**).

**Figure 6:**
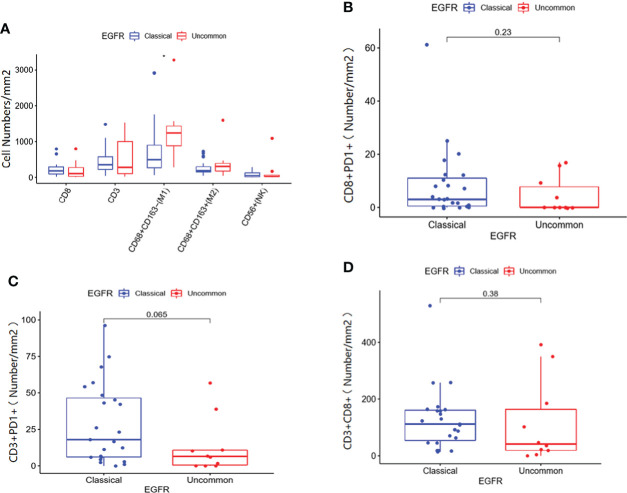
Immune cell infiltration in patients with classical EGFR mutations vs. uncommon EGFR mutations in clinical center data. **(A)** Comparison of immune cells in NSCLC patients from our clinical center between EGFR classical mutations and uncommon mutations. The infiltration of CD8 + PD1 + cells **(B)**, CD3 + PD1 + cells **(C)**, and CD3 + CD8 + cells **(D)** in patients with EGFR classical mutations and uncommon mutations.

Immune cell infiltration in patients with EGFR mutations within clinical center data were shown in **Figure 6**. According to infiltration of immune cells (**Supplementary Figure 1**), M1 macrophages in uncommon EGFR mutated tumor samples was higher than that in classical EGFR mutated tumor samples (**Figure 6A**) (P < 0.05). No statistically significant differences in the infiltration of CD3 + T cells, CD8 + T cells, M2 macrophages, and NK cells were observed between uncommon EGFR mutations and classical EGFR mutations (**Figure 6A**). The infiltration of CD8 + PD1 + cells (P = 0.23), CD3 + PD1 + cells (P = 0.065), and CD3 + CD8 + cells (P = 0.38) were also evaluated but no statistically significant differences were observed between uncommon and classical EGFR mutations (**Figures 6B-D**).

### Prognosis of EGFR-mutated NSCLC patients with different infiltrations of immune cells

**Figure 7:**
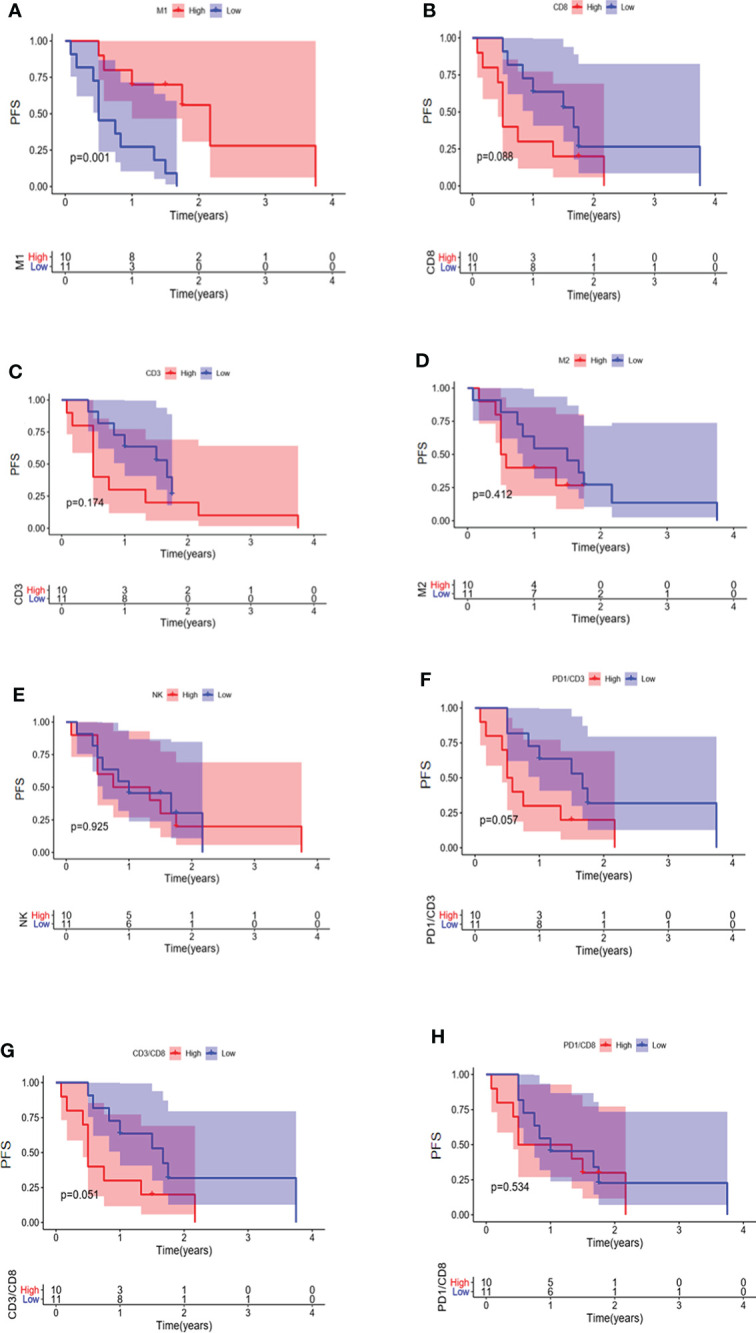
PFS in EGFR-mutated NSCLC patients with different infiltrations of immune cells. Comparison of patient PFS between high and low infiltration of M1 macrophages **(A)**, CD8 + T cells **(B)**, CD3 + T cells **(C)**, M2 macrophages **(D)**, NK cells **(E)**, CD3 + PD1 + T cells **(F)**, CD3 + CD8 + T cells **(G)**, and CD8 + PD1 + T cells **(H)**.

The relationship between immune cell infiltration and prognosis was further analyzed in 33 EGFR mutated NSCLC patients. Our results indicated that the PFS of patients with higher infiltration of M1 macrophages was longer than the low infiltration group (**Figure 7A**) (P = 0.001). No significant association was observed between patient prognosis and infiltration of CD8 + T cells, CD3 + T cells, M2 macrophages, NK cells, CD3 + PD1 + T cells, CD3 + CD8 + T cells, and CD8 + PD1 + T cells (**Figures 7B-H**).

### Uncommon EGFR mutated NSCLC patients who benefited from an immune checkpoint inhibitor

**Figure 8:**
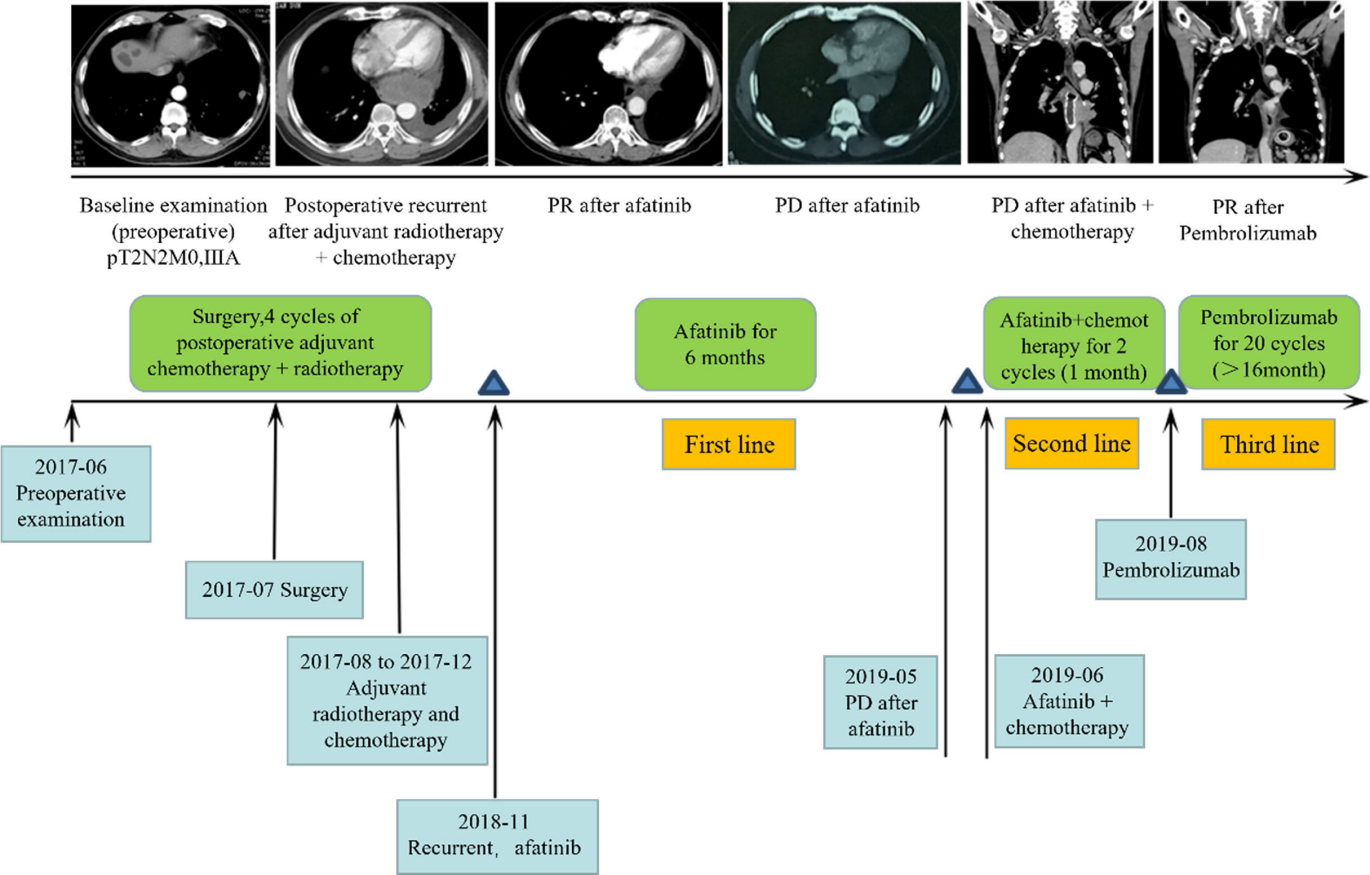
Clinical response for third-line immunotherapy in a patient with uncommon type EGFR mutation.

To evaluate the treatment benefits of immune checkpoint inhibitors for NSCLC patients with uncommon EGFR mutations, we reported an NSCLC patient with uncommon EGFR mutation who benefited from immunotherapy. The 59-year-old male patient with smoking history for more than 40 years was pathologically diagnosed as adenosquamous carcinoma (T2N2M0IIIA). Uncommon EGFR mutations, including G719C and S768I, were identified in tumor sample. The patient received 20 cycles of Pembrolizumab (PD-1 inhibitor) treatment (**Figure 8**). During treatment, the patient maintained partial response (PR), and the primary and metastatic lesions were controlled for more than 16 months. At present, the patient continues to receive Pembrolizumab. The immune cell infiltration in the tumor tissue of the patient was further detected. The density of M1 macrophages was higher than the median level of the above classical EGFR mutation (2515/mm^2^ vs. 854/mm^2^).

## Discussion

In this retrospective analysis, we revealed a correlation between EGFR mutations and immunotherapy biomarkers. Our results indicated differences in PD-L1, TMB, and tumor immune cell infiltration between NSCLC patients with and without EGFR mutations. Differences in TMB and immune cell infiltration amongst subtypes of EGFR mutations were also investigated. We used clinical samples of NSCLC as a validation cohort in order to explore PD-L1, TMB, and immune cell infiltration in patients harboring different subtypes of EGFR mutations. Our results indicate that TMB and PD-L1 were lower in NSCLC patients with EGFR mutations than in wild-type. Further analyses in subtypes of EGFR mutations indicated that the expression of PD-L1, TMB, and the proportion of TMB-H/PD-L1+ were higher in patients with uncommon EGFR mutations, especially the G719x mutation, than classical EGFR mutations. TIME analyses indicated that the infiltration of M1 macrophages was higher in patients with uncommon EGFR mutations than classical mutations. Our study further explored differences in TMB, PD-L1, and immune cell infiltration amongst different subtypes of EGFR mutations.

PD-L1 is a biomarker that can predict the efficacy of PD-1/PD-L1 inhibitors for NSCLC patients. At the cellular level, it was found that activation of the EGFR pathway in bronchial epithelial cells can induce the expression of PD-L1 in tumor cells (22). The expression of PD-L1 was higher in EGFR-mutated NSCLC cells than in wild-type (23). Previous studies on tumor tissue of NSCLC also suggested that the expression of PD-L1 was higher in EGFR mutated NSCLC than wild-type (23, 24). In contrast, other studies have indicated that the positive expression of PD-L1 was lower in EGFR mutated patients than in wild-type (24, 25). The results of our study are consistent with the latter studies.

Various factors could impact PD-L1 expression. IFN-γ secreted by activated T cells can induce tumor cells to express PD-L1, which indicates that expression of PD-L1 can be regulated by TIME-related factors. The TIME status should be further characterized in tissues rather than in single tumor cells. Additionally, in tumors, the expression of PD-L1 can be regulated at pathway level through carcinogenic signals, gene mutations, microRNA-based controls, and post-translational regulations. PD-L1 expression in NSCLC tumor cells can be promoted by the mTOR and ERK regulatory mechanisms in activated EGFR pathways. Factors mediating EGFR signaling and PD-L1 expression, such as NF-k BP65, STAT3, and/or JAK2-STAT1, also play important roles (26). In this study, the infiltration of CD8 + T cells in EGFR mutated NSCLC was lower than in the EGFR wild-type. Given that IFN-γ secreted by CD8 + T cells can regulate the expression of PD-L1, we speculate that the different expression of PD-L1 between EGFR wild-type and EGFR mutated NSCLC is related to the infiltration of CD8 + T cells, in addition to EGFR activation pathways. In EGFR wild-type NSCLC, highly infiltrated CD8 + T cells can secrete a large amount of IFN-γ, further promoting the expression of PD-L1 in tumor cells. In contrast, low infiltration of CD8 + T cells in EGFR mutated NSCLC leads to the low expression of PD-L1.

The study showed that the expression of PD-L1 in uncommon and classical EGFR mutations was different between TCGA database data and clinical center data, which may due to the differences in sample size and detection methods. More EGFR-mutated patients were included in the clinical center data than in the TCGA database data. In addition, compared with the TCGA database, the clinical center adopted one method to detect PD-L1, which led to more comparability of PD-L1 detection results among EGFR subgroups in clinical center data than in TCGA database data.

TMB is another biomarker for predicting the efficacy of PD-1/PD-L1 inhibitors in NSCLC. Non-synonymous mutations can change amino acid sequences and promote the production of new antigens in order to promote an immune response (27). EGFR mutated NSCLC cells have fewer non-synonymous mutations and therefore produce fewer new antigens. Several studies have indicated that TMB in EGFR mutated NSCLC is lower than in EGFR wild-type (28). Compared with previous findings, we also obtained consistent results on TMB.

Low TMB in patients with EGFR mutations may be attributed to smoking status. Somatic cell mutation is a gradual accumulation process, and the number of mutations is proportional to age (29). A study indicated that TMB increases with age, with TMB in patients aged 90 at a level 2.4 times higher than TMB in patients aged 10 (30). Multivariate analyses in our study indicated that the EGFR G719x mutation is more likely to occur in elderly patients than the EGFR ex19del mutation (16). We speculate that the difference in TMB between uncommon and classical EGFR mutations may be related to differences in age. Several studies have shown that TMB is higher in LRP1B mutated patients than in wild-type patients (31, 32). Patients harboring LRP1B mutation are more likely to benefit from PD-1/PD-L1 inhibitors (32). We identified co-mutated genes in different subtypes of EGFR mutations and found that patients with G719x mutation showed the highest frequency of co-mutation with LRP1B. The presence of LRP1B mutation may be one of the reasons for the high TMB of G719X mutated NSCLC. The result supports the view that NSCLC patients with uncommon EGFR mutations may benefit from PD-1/PD-L1 inhibitors.

The essence of immune checkpoint inhibitors is to restart anti-tumor immunity by inhibiting tumor immune escape and restoring the anti-tumor activity of T cells. The infiltration of immune cells in tumors plays a key role in the efficacy of immunotherapy. NSCLC patients with high infiltration of CD8 + T cells tend to benefit from immune checkpoint inhibitors (33). The TIME of EGFR mutated NSCLC lacks the infiltration of T cells and PD-L1 +/CD8 + T cells; therefore was classified as an immune desert (25). Indeed, our study observed that CD8 + T cell infiltration in EGFR mutated NSCLC is lower than wild-type.

We additionally found that activation of CD4 + T cells and M1 macrophages can promote anti-tumor immunity and have low infiltration in EGFR mutated NSCLC, which could limit the killing efficiency of CD8 + T cells. In this study, no statistically significant difference in the infiltration of immune cells between patients with uncommon EGFR mutations and EGFR wild-type was observed. In contrast, the infiltration of CD8 + T cells and activated CD4 + T cells in patients with classical EGFR mutations was lower than those in the EGFR wild-type. The results suggest that some subgroups of patients with high infiltration of immune cells might be masked during TIME analysis of all EGFR mutated NSCLC. Further analysis of subgroups of EGFR mutations is necessary.

We found that the infiltration of M1 macrophages in patients with uncommon EGFR mutations was higher than that in classical EGFR mutations. Patients with higher infiltration of M1 macrophages have more favorable prognosis than low infiltration of M1 macrophages. M1 macrophages play an important role in anti-tumor immunity. In the adaptive immune process, M1 macrophages can provide defense and kill tumor cells by secreting pro-inflammatory factors and reactive oxygen species/nitrogen. In the specific immune process (34), M1 can promote anti-tumor response of T cells under tumor-specific antigen stimulation. Studies have shown that individuals with high M1/M2 are less likely to develop tumors. Depolarizing M2 macrophages and repolarizing them into M1 macrophages can prevent the growth of cancer cells (35). Rao et al. (36) detected the TIME of malignant glioma mice lacking CD8 + T cells that could benefit from PD-1 inhibitors, and determined that a large amount of M1 macrophages were present in TIME. PD-1 expressed in macrophages can inhibit the phagocytosis of macrophages (37). We speculate that PD-1 inhibitors may promote the phagocytosis of macrophages by blocking PD-1 in order to enhance anti-tumor immunity. Therefore, the difference in the infiltration of M1 macrophages may be one of the reasons for differences in the efficacy of PD-1/PD-L1 inhibitors in patients with uncommon and classical EGFR mutations.

The efficacy of immunotherapy is not only related to the expression of PD-L1, TMB, and immune cell infiltration but also related to the types of gene mutations and co-mutated genes (17, 19, 38–40). A large number of studies have shown that integrating immunotherapy with different biomarkers improves the accuracy of prediction (41). Therefore, the response of EGFR mutated NSCLC to immunotherapy is jointly predicted by different biomarkers, rather than a single predictor.

There were some limitations in our study. First, varies treatment methods during the early stage had different impacts on TMB and PD-L1. However, the 1,111 patients with NSCLC in our study were not selected according to different treatment schemes in the early stage. Second, the size of the clinical sample is small in the context of studying the characteristics of TIME. Finally, our study did not explore the prognosis of immunotherapy in patients with different TMB, PD-L1, and immune cell infiltration.

Our study revealed TMB, PD-L1, and immune cell infiltration in different subtypes of EGFR mutations. The findings of our study can provide a theoretical reference for selecting EGFR mutated NSCLC patients who would likely to receive immunotherapy. Further studies on the efficacy of PD-1/PD-L1 inhibitors in patients with EGFR subtype mutations are continuously being undertaken.

## Data availability statement

The original contributions presented in the study are included in the article/**Supplementary Material**. Further inquiries can be directed to the corresponding author.

## Author contributions

YS, TM and JJ designed the study. TM, JJ, RH, XL and GF collected the samples with clinical information. TM, JJ, QZ, WL, XH, CX and YW contributed to the analysis and interpretation of the data. YS, TM and JJ wrote the draft of the manuscript. All authors reviewed and approved the final version of the manuscript.

## Funding

This work was supported by Government-funded Medical Talent Training Program of Hebei Province in 2020.

## Conflict of interest

The authors declare that the research was conducted in the absence of any commercial or financial relationships that could be construed as a potential conflict of interest.

## Publisher’s note

All claims expressed in this article are solely those of the authors and do not necessarily represent those of their affiliated organizations, or those of the publisher, the editors and the reviewers. Any product that may be evaluated in this article, or claim that may be made by its manufacturer, is not guaranteed or endorsed by the publisher.

## References

[1] Ferlay J Steliarova-Foucher E Lortet-Tieulent J Rosso S Coebergh JW Comber H. Cancer incidence and mortality patterns in Europe: estimates for 40 countries in 2012. Eur J Cancer (2013) 49:1374–403. doi: 10.1016/j.ejca.2012.12.027 23485231

[2] Torre LA Bray F Siegel RL Ferlay J Lortet-Tieulent J Jemal A. Global cancer statistics, 2012. CA Cancer J Clin (2015) 65:87–108. doi: 10.3322/caac.21262 25651787

[3] Chan BA Hughes BG. Targeted therapy for non-small cell lung cancer: current standards and the promise of the future. Transl Lung Cancer Res (2015) 4(1):36–54. doi: 10.3978/j.issn.2218-6751 25806345 PMC4367711

[4] Zhou C Wu Y-L Chen G Feng J Liu X-Q Wang C. Erlotinib versus chemotherapy as first-line treatment for patients with advanced EGFR mutation-positive non-small-cell lung cancer (OPTIMAL, CTONG-0802): a multicentre, open-label, randomised, phase 3 study. Lancet Oncol (2011) 12:735–42. doi: 10.1016/S1470-2045(11)70184-X 21783417

[5] Rosell R Carcereny E Gervais R Vergnenegre A Massuti B Felip E. Erlotinib versus standard chemotherapy as first-line treatment for European patients with advanced EGFR mutation-positive non-small-cell lung cancer (EURTAC): a multicentre, open-label, randomised phase 3 trial. Lancet Oncol (2012) 13:239–46. doi: 10.1016/S1470-2045(11)70393-X 22285168

[6] Goss G Tsai C-M Shepherd FA Bazhenova L Lee JS Chang G-C. Osimertinib for pretreated EGFR Thr790Met-positive advanced non-small-cell lung cancer (AURA2): a multicentre, open-label, single-arm, phase 2 study. Lancet Oncol (2016) 17:1643–52. doi: 10.1016/S1470-2045(16)30508-3 27751847

[7] Mok TS Wu YL Thongprasert S Yang CH Chu DT Saijo N. Gefitinib or carboplatin-paclitaxel in pulmonary adenocarcinoma. N Engl J Med (2009) 361(10):947–57. doi: 10.1056/NEJMoa0810699 19692680

[8] Schuler M Wu YL Hirsh V O'Byrne K Yamamoto N Mok T. First-line afatinib versus chemotherapy in patients with non-small cell lung cancer and common epidermal growth factor receptor gene mutations and brain metastases. J Thorac Oncol (2016) 11:380–90. doi: 10.1016/j.jtho.2015.11.014 26823294

[9] Park K Tan E-H O'Byrne K Zhang L Boyer M Mok T. Afatinib versus gefitinib as first-line treatment of patients with EGFR mutation-positive non-small-cell lung cancer (LUX-lung 7): a phase 2B, open-label, randomised controlled trial. Lancet Oncol (2016) 17:577–89. doi: 10.1016/S1470-2045(16)30033-X 27083334

[10] Soria JC Ohe Y Vansteenkiste J Reungwetwattana T Chewaskulyong B Lee KH. Osimertinib in untreated EGFR-mutated advanced non-Small-Cell lung cancer. N Engl J Med (2018) 378:113–25. doi: 10.1056/NEJMoa1713137 29151359

[11] Fehrenbacher L Spira A Ballinger M Kowanetz M Vansteenkiste J Mazieres J. Atezolizumab versus docetaxel for patients with previously treated non-small-cell lung cancer (POPLAR): a multicentre, open-label, phase 2 randomised controlled trial. Lancet (2016) 387:1837–46. doi: 10.1016/S0140-6736(16)00587-0 26970723

[12] Schmid P Adams S Rugo HS Schneeweiss A Barrios CH Iwata H. Atezolizumab and nab-paclitaxel in advanced triple-negative breast cancer. N Engl J Med (2019) 380:985–8. doi: 10.1056/NEJMc1900150 30345906

[13] Gettinger S Hellmann MD Chow LQM Borghaei H Antonia S Brahmer JR. Nivolumab plus erlotinib in patients with EGFR-mutant advanced NSCLC. J Thorac Oncol (2018) 13:1363–72. doi: 10.1016/j.jtho.2018.05.015 29802888

[14] Oxnard GR Yang JC Yu H Kim SW Saka H Horn L. TATTON: a multi-arm, phase ib trial of osimertinib combined with selumetinib, savolitinib, or durvalumab in EGFR-mutant lung cancer. Ann Oncol (2020) 31:507–16. doi: 10.1016/j.annonc.2020.01.013 32139298

[15] Socinski MA Jotte RM Cappuzzo F Orlandi F Stroyakovskiy D Nogami N. Atezolizumab for first-line treatment of metastatic nonsquamous NSCLC. N Engl J Med (2018) 378:2288–301. doi: 10.1056/NEJMoa1716948 29863955

[16] Chen K Cheng G Zhang F Zhu G Xu Y Yu X. PD-L1 expression and T cells infiltration in patients with uncommon EGFR-mutant non-small cell lung cancer and the response to immunotherapy. Lung Cancer (2020) 142:98–105. doi: 10.1016/j.lungcan.2020.02.010 32120230

[17] Yoshida H Kim YH Ozasa H Nagai H Sakamori Y Tsuji T. Nivolumab in non-small-cell lung cancer with EGFR mutation. Ann Oncol (2018) 29:777–8. doi: 10.1093/annonc/mdx745 29161357

[18] Barlesi F Vansteenkiste J Spigel D Ishii H Garassino M de Marinis F. Avelumab versus docetaxel in patients with platinum-treated advanced non-small-cell lung cancer (JAVELIN lung 200): an open-label, randomised, phase 3 study. Lancet Oncol (2018) 19:1468–79. doi: 10.1016/S1470-2045(18)30673-9 30262187

[19] Yamada T Hirai S Katayama Y Yoshimura A Shiotsu S Watanabe S. Retrospective efficacy analysis of immune checkpoint inhibitors in patients with EGFR-mutated non-small cell lung cancer. Cancer Med (2019) 8:1521–9. doi: 10.1002/cam4.2037 PMC6488155 30790471

[20] Reck M Rodríguez-Abreu D Robinson AG Hui Reck Csőszi T Fülöp A. Pembrolizumab versus chemotherapy for PD-L1-Positive non-Small-Cell lung cancer. N Engl J Med (2016) 375(19):1823. doi: 10.1056/NEJMoa1606774 27718847

[21] Herbst RS Baas P Kim DW Felip E Pérez-Gracia JL Han JY. Pembrolizumab versus docetaxel for previously treated, PD-L1-positive, advanced non-small-cell lung cancer (KEYNOTE-010): a randomised controlled trial. Lancet (2015) 387(10027):1540–50. doi: 10.1016/S0140-6736(15)01281-7 26712084

[22] Akbay EA Koyama S Carretero J Altabef A Tchaicha JH Christensen CL. Activation of the PD-1 pathway contributes to immune escape in EGFR-driven lung tumors. Cancer Discov (2013) 3:1355–63. doi: 10.1158/2159-8290.CD-13-0310 PMC3864135 24078774

[23] D'Incecco A Andreozzi M Ludovini V Rossi E Capodanno A Landi L. PD-1 and PD-L1 expression in molecularly selected non-small-cell lung cancer patients. Br J Cancer (2015) 112:95–102. doi: 10.1038/bjc.2014.555 25349974 PMC4453606

[24] Soo RA Lim SM Syn NL Teng R Soong R Mok TSK. Immune checkpoint inhibitors in epidermal growth factor receptor mutant non-small cell lung cancer: Current controversies and future directions. Lung Cancer (2018) 115:12–20. doi: 10.1016/j.lungcan.2017.11.009 29290252

[25] Dong ZY Zhang JT Liu SY Su J Zhang C Xie Z. EGFR mutation correlates with uninflamed phenotype and weak immunogenicity, causing impaired response to PD-1 blockade in non-small cell lung cancer. Oncoimmunology (2017) 6:e1356145. doi: 10.1080/2162402X.2017.1356145 29147605 PMC5674946

[26] Malhotra J Jabbour SK Aisner J. Current state of immunotherapy for non-small cell lung cancer. Transl Lung Cancer Res (2017) 6:196–211. doi: 10.21037/tlcr.2017.03.01 28529902 PMC5420529

[27] Hellmann MD Ciuleanu TE Pluzanski A Lee JS Otterson GA Audigier-Valette C. Nivolumab plus ipilimumab in lung cancer with a high tumor mutational burden. N Engl J Med (2018) 378:2093–104. doi: 10.1056/NEJMoa1801946 PMC7193684 29658845

[28] Santaniello A Napolitano F Servetto A De Placido P Silvestris N Bianco C. Tumour microenvironment and immune evasion in EGFR addicted NSCLC: Hurdles and possibilities. Cancers (Basel) (2019) 11:1419. doi: 10.3390/cancers11101419 PMC6826622 31554160

[29] Alexandrov LB Jones PH Wedge DC Sale JE Campbell PJ Nik-Zainal S. Clock-like mutational processes in human somatic cells. Nat Genet (2015) 47:1402–7. doi: 10.1038/ng.3441 PMC4783858 26551669

[30] Chalmers ZR Connelly CF Fabrizio D Gay L Ali SM Ennis R. Analysis of 100,000 human cancer genomes reveals the landscape of tumor mutational burden. Genome Med (2017) 9:34. doi: 10.1186/s13073-017-0424-2 28420421 PMC5395719

[31] Lan S Li H Liu Y Ma L Liu X Liu Y. Somatic mutation of LRP1B is associated with tumor mutational burden in patients with lung cancer. Lung Cancer (2019) 132:154–6. doi: 10.1016/j.lungcan.2019.04.025 31054778

[32] Chen H Chong W Wu Q Yao Y Mao M Wang X. Association of LRP1B mutation with tumor mutation burden and outcomes in melanoma and non-small cell lung cancer patients treated with immune check-point blockades. Front Immunol (2019) 10:1113. doi: 10.3389/fimmu.2019.01113 31164891 PMC6536574

[33] Hu-Lieskovan S Lisberg A Zaretsky JM Grogan TR Rizvi H Wells DK. Tumor characteristics associated with benefit from pembrolizumab in advanced non-small cell lung cancer. Clin Cancer Res (2019) 25:5061–8. doi: 10.1158/1078-0432.CCR-18-4275 PMC6901027 31113840

[34] Aras S Zaidi MR. TAMeless traitors: macrophages in cancer progression and metastasis. Br J Cancer (2017) 117:1583–91. doi: 10.1038/bjc.2017.356 PMC5729447 29065107

[35] Mills CD Lenz LL Harris RA. A breakthrough: Macrophage-directed cancer immunotherapy. Cancer Res (2016) 76:513–6. doi: 10.1158/0008-5472.CAN-15-1737 PMC4738030 26772756

[36] Rao G Latha K Ott M Sabbagh A Marisetty A Ling X. Anti-PD-1 induces M1 polarization in the glioma microenvironment and exerts therapeutic efficacy in the absence of CD8 cytotoxic T cells. Clin Cancer Res (2020) 26:4699–712. doi: 10.1158/1078-0432.CCR-19-4110 PMC7483850 32554515

[37] Gordon SR Maute RL Dulken BW Hutter G George BM McCracken MN. PD-1 expression by tumour-associated macrophages inhibits phagocytosis and tumour immunity. Nature (2017) 545:495–9. doi: 10.1038/nature22396 PMC5931375 28514441

[38] Hastings K Yu HA Wei W Sanchez-Vega F DeVeaux M Choi J. EGFR mutation subtypes and response to immune checkpoint blockade treatment in non-small-cell lung cancer. Ann Oncol (2019) 30(8):1311–20. doi: 10.1093/annonc/mdz141 PMC6683857 31086949

[39] Bai X Wu DH Ma SC Wang J Tang XR Kang S. Development and validation of a genomic mutation signature to predict response to PD-1 inhibitors in non-squamous NSCLC: a multicohort study. J Immunother Cancer (2020) 8:e000381. doi: 10.1136/jitc-2019-000381 32606052 PMC7328897

[40] Dong ZY Zhong WZ Zhang XC Su J Xie Z Liu SY. Potential predictive value of TP53 and KRAS mutation status for response to PD-1 blockade immunotherapy in lung adenocarcinomas. Clin Cancer Res (2017) 23(12):3012–24. doi: 10.1158/1078-0432.CCR-16-2554 28039262

[41] Camidge DR Doebele RC Kerr KM. Comparing and contrasting predictive biomarkers for immunotherapy and targeted therapy of NSCLC. Nat Rev Clin Oncol (2019) 16:341–55. doi: 10.1038/s41571-019-0173-9 30718843

