# Supplementary material for: PD-L1 expression, tumor mutational burden, and immune cell infiltration in non-small cell lung cancer patients with epithelial growth factor receptor mutations
Source: Front Oncol. 2022 Aug 5;12:922899. doi: 10.3389/fonc.2022.922899 (PMC9389166; doi:10.3389/fonc.2022.922899)
Supplement: Supplementary file 1 [file Presentation_1.pptx]

## Slide 1
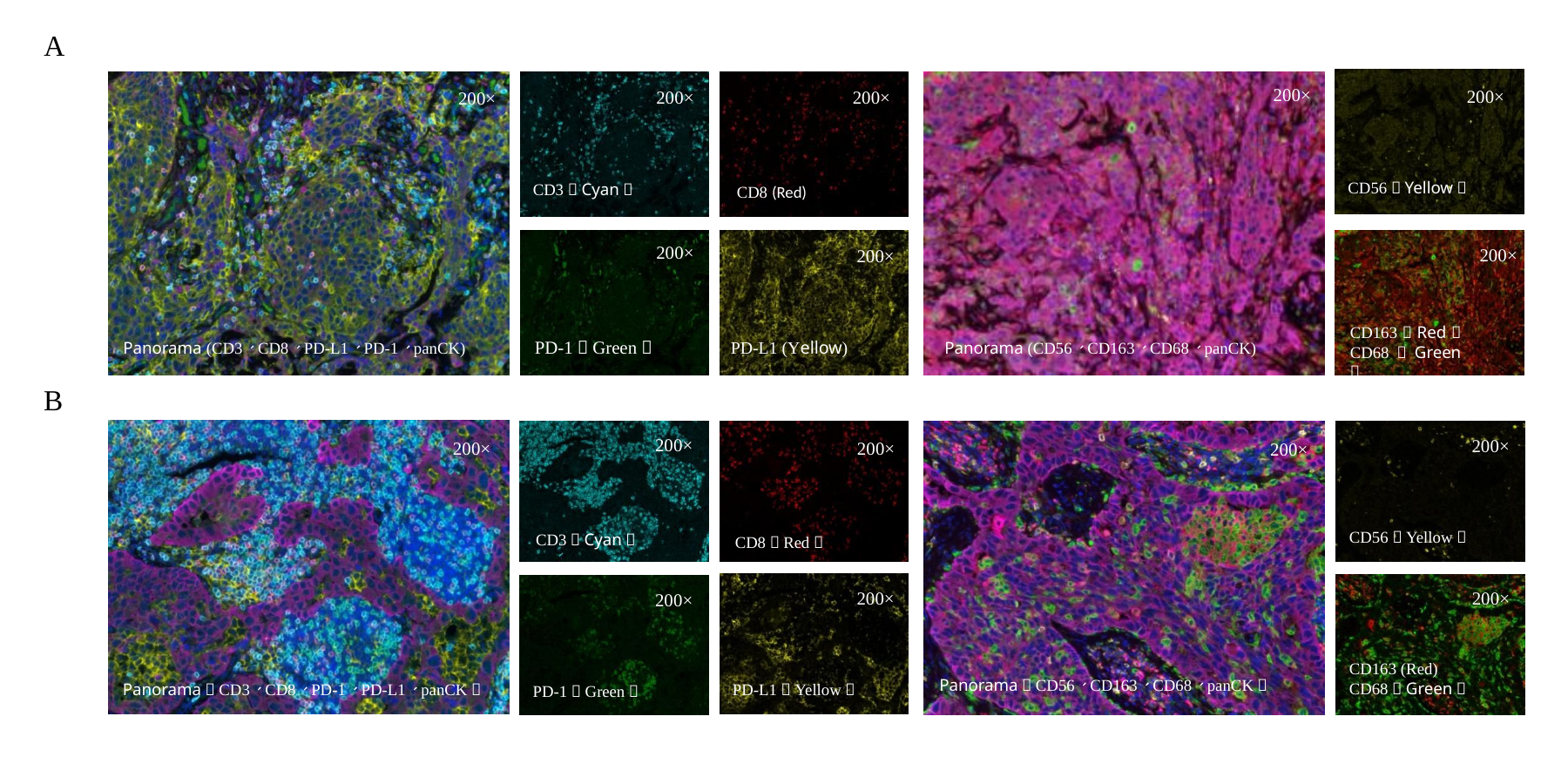

A
200×
200×
200×
200×
200×
CD56（Yellow）
CD3（Cyan）
CD8 (Red)
200×
200×
200×
CD163（Red）CD68（Green）
PD-1（Green）
Panorama (CD3、CD8、PD-L1、PD-1、panCK)
PD-L1 (Yellow)
Panorama (CD56、CD163、CD68、panCK)
B
200×
200×
200×
200×
200×
CD56（Yellow）
CD3（Cyan）
CD8（Red）
200×
200×
200×
CD163 (Red)
CD68（Green）
Panorama（CD56、CD163、CD68、panCK）
PD-L1（Yellow）
Panorama（CD3、CD8、PD-1、PD-L1、panCK）
PD-1（Green）
