# Supplementary material for: PD-L1 expression, tumor mutational burden, and immune cell infiltration in non-small cell lung cancer patients with epithelial growth factor receptor mutations
Source: Front Oncol. 2022 Aug 5;12:922899. doi: 10.3389/fonc.2022.922899 (PMC9389166; doi:10.3389/fonc.2022.922899)
Supplement: Supplementary file 2 [file Table_1.doc]

Supplementary Table 1

Baseline characteristics of patients with EGFR mutation and EGFR wild type from the TCGA dataset

| Characteristic | Total (N=474) | EGFR-Wild (N=423) | EGFR-alteration (N=51) | Mutation rate | P-value |
| --- | --- | --- | --- | --- | --- |
| Gender |  |  |  |  | 0.137 |
| male | 216 | 198 | 18 | 8.3% |  |
| female | 258 | 225 | 33 | 12.8% |
| Age |  |  |  |  | 0.881 |
| ＜65 | 205 | 182 | 23 | 11.2% |  |
| ≥65 | 269 | 241 | 28 | 10.4% |
| Stage |  |  |  |  | 0.928 |
| Ⅰ | 260 | 237 | 23 | 8.8% |  |
| Ⅱ | 116 | 104 | 12 | 10.3% |
| Ⅲ | 177 | 163 | 14 | 7.9% |
| Ⅳ | 21 | 19 | 2 | 9.5% |
| T |  |  |  |  | 0.512 |
| T1 | 160 | 144 | 16 | 10.0% |  |
| T2 | 255 | 228 | 27 | 12.0% |  |
| T3 | 40 | 36 | 4 | 10.0% | 0.09 |
| N |  |  |  |  |  |
| N0 | 110 | 83 | 27 | 24.5% |  |
| N1 | 87 | 78 | 9 | 10.3% |
| N2 | 68 | 56 | 12 | 17.6% |
| N3 | 2 | 1 | 1 | 50.0% |
| Nx | 7 | 5 | 2 | 28.6% |
| M |  |  |  |  | 0.771 |
| M0 | 330 | 292 | 38 | 11.5% |  |
| M1 | 21 | 19 | 2 | 9.5% |
| Mx | 123 | 112 | 11 | 8.9% |

Supplementary Table 2

Baseline characteristics of patients with subtypes of EGFR mutations and EGFR wild type

| Characteristic | EGFR-Wild  (N=423) | EGFR-alteration | | P-value |
| --- | --- | --- | --- | --- |
|  |  | Classical mutation  (N=17) | Uncommon  (N=17) |  |
| Gender |  |  |  | 0.065 |
| male | 198 | 3 | 8 |  |
| female | 225 | 14 | 9 |  |
| Age |  |  |  | 1.000 |
| ＜65 | 182 | 7 | 7 |  |
| ≥65 | 241 | 10 | 10 |  |
| Stage |  |  |  | 0.749 |
| Ⅰ | 237 | 7 | 9 |  |
| Ⅱ | 104 | 4 | 5 |  |
| Ⅲ | 163 | 5 | 3 |  |
| Ⅳ | 19 | 1 | 0 |  |
| T |  |  |  | 0.525 |
| T1 | 144 | 6 | 6 |  |
| T2 | 228 | 9 | 7 |  |
| T3 | 36 | 2 | 2 |  |
| T4 | 15 | 0 | 2 |  |
| N |  |  |  | 0.234 |
| N0 | 83 | 7 | 11 |  |
| N1 | 78 | 4 | 3 |  |
| N2 | 56 | 5 | 2 |  |
| N3 | 1 | 0 | 0 |  |
| Nx | 5 | 1 | 1 |  |
| M |  |  |  | 0.306 |
| M0 | 292 | 15 | 13 |  |
| M1 | 19 | 1 | 0 |  |
| Mx | 112 | 1 | 4 |  |

Supplementary Table 3

Baseline clinical data of 33 NSCLC patients with EGFR mutation

| Characteristic | Total (N=33) | Uncommon (N=10) | Classical mutation (N=23) | P-value |
| --- | --- | --- | --- | --- |
| Gender |  |  |  |  |
| Male | 14 (42.4%) | 6 (60.0%) | 8 (34.8%) | 0.257 |
| Female | 19 (57.6%) | 4 (40.0%) | 15 (65.2%) |
| Age |  |  |  |  |
| ＜65 | 13 (39.4%) | 6 (60.0%) | 7 (30.4%) | 0.139 |
| ≥65 | 20 (60.6%) | 4 (40.0%) | 16 (69.6%) |
| Stage |  |  |  |  |
| Ⅰ | 3 (9.1%) | 0 (0.0%) | 3 (13.0%) | 0.128 |
| Ⅱ | 1 (3.0%) | 1 (10.0%) | 0 (0.0%) |
| Ⅲ | 5 (15.2%) | 3 (30.0%) | 2 (8.7%) |
| Ⅳ | 24 (72.7%) | 6 (60.0%) | 18 (78.3%) |
| Surgery |  |  |  |  |
| Yes | 6 (18.2%) | 2 (20.0%) | 5 (21.7%) | 1.000 |
| No | 27 (81.8%) | 8 (80.0%) | 18 (78.3%) |
| Smoke |  |  |  |  |
| Yes | 10 (30.3%) | 2 (20.0%) | 8 (34.8%) | 0.140 |
| No | 17 (51.5%) | 4 (40.0%) | 13 (56.5%) |
| Ever | 6 (18.2%) | 4 (40.0%) | 2 (8.7%) |
| Pathological |  |  |  |  |
| Adenocarcinoma | 32 (97.0%) | 9 (90.0%) | 23 (100.0%) | 0.303 |
| Others | 1 (3.0%) | 1 (10.0%) | 0 (0.0%) |
| Treatment |  |  |  |  |
| First-line  (targeted therapy) | 12 (36.4%) | 4 | 8 | 0.718 |
| ≥Second line | 11 (33.3%) | 4 | 7 |
| targeted therapy | 9 (27.3%) | 3 | 6 |
| Immunotherapy | 2 (6.1%) | 1 | 1 |
| Untreated | 10 (30.3%) | 2 | 8 |
